# Collagen Fiber Orientation and Dispersion in the Upper Cervix of Non-Pregnant and Pregnant Women

**DOI:** 10.1371/journal.pone.0166709

**Published:** 2016-11-29

**Authors:** Wang Yao, Yu Gan, Kristin M. Myers, Joy Y. Vink, Ronald J. Wapner, Christine P. Hendon

**Affiliations:** 1 Department of Mechanical Engineering, Columbia University, New York, NY, United States of America; 2 Department of Electrical Engineering, Columbia University, New York, NY, United States of America; 3 Department of Obstetrics and Gynecology, Columbia University Medical Center, New York, NY, United States of America; Massey University, NEW ZEALAND

## Abstract

The structural integrity of the cervix in pregnancy is necessary for carrying a pregnancy until term, and the organization of human cervical tissue collagen likely plays an important role in the tissue’s structural function. Collagen fibers in the cervical extracellular matrix exhibit preferential directionality, and this collagen network ultrastructure is hypothesized to reorient and remodel during cervical softening and dilation at time of parturition. Within the cervix, the upper half is substantially loaded during pregnancy and is where the premature funneling starts to happen. To characterize the cervical collagen ultrastructure for the upper half of the human cervix, we imaged whole axial tissue slices from non-pregnant and pregnant women undergoing hysterectomy or cesarean hysterectomy respectively using optical coherence tomography (OCT) and implemented a pixel-wise fiber orientation tracking method to measure the distribution of fiber orientation. The collagen fiber orientation maps show that there are two radial zones and the preferential fiber direction is circumferential in a dominant outer radial zone. The OCT data also reveal that there are two anatomic regions with distinct fiber orientation and dispersion properties. These regions are labeled: Region 1—the posterior and anterior quadrants in the outer radial zone and Region 2—the left and right quadrants in the outer radial zone and all quadrants in the inner radial zone. When comparing samples from nulliparous vs multiparous women, no differences in these fiber properties were noted. Pregnant tissue samples exhibit an overall higher fiber dispersion and more heterogeneous fiber properties within the sample than non-pregnant tissue. Collectively, these OCT data suggest that collagen fiber dispersion and directionality may play a role in cervical remodeling during pregnancy, where distinct remodeling properties exist according to anatomical quadrant.

## Introduction

**Fig 1:**
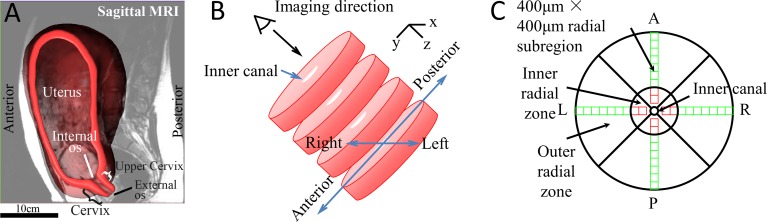
The anatomical position of the cervix, sample preparation and terms. (A) The anatomical position of the uterus and the cervix produced from an MRI of a patient at 22 weeks of gestation [4]. The proximal end of the cervix is the internal os and the distal end of the cervix is the external os. (B) Illustration of specimen preparation of the upper cervix. The cervix is cut perpendicular to the inner canal to obtain slices for experiments. The sliced cervix in (B) is oriented the same as shown in (A). The imaging direction is perpendicular to cutting direction, normal to cervical slices. (C) Illustration of four anatomical quadrants (A-anterior, P-posterior, L-left, and R-right), inner and outer radial zones, and 400μm × 400μm subregions used for fiber dispersion analysis.

The cervix is a dense fibrous tissue that is located at the lowest part of the uterus (Fig 1). It is cylindrical in shape with average dimensions of 3 cm long and 2.5 cm in diameter[1]. The mechanical function of the cervix is crucial for a term pregnancy (defined as a pregnancy that extends beyond 37 weeks of gestation). Cervical mechanical function has two roles: 1) prior to term it must remain closed and resist the increasing mechanical load from the growing pregnancy and 2) at time of parturition it must be soft to deform and dilate to allow for delivery of the fetus. To accommodate this drastic dilation of the cervix at time of delivery, the extracellular matrix (ECM) of the tissue must drastically remodel, reorganize, and soften during gestation. The timing and characteristics of this remodeling behavior is currently an active research focus because it is hypothesized that premature remodeling in pregnancy can lead to a preterm birth [2], a leading cause of neonatal death or significant neonatal morbidity [3]. In an effort to characterize the remodeling behavior of human cervical tissue, the objective of this study is to measure and quantify the collagen fiber orientation and dispersion (e.g. ultrastructure) of non-pregnant and term pregnant cervical tissue using optical coherence tomography (OCT).

The premature change in mechanical properties of the cervix induced by alterations of cervical tissue ECM content and ultrastructure is thought to contribute to cervical failure leading to preterm birth. Cervical collagen exists as fibers in a hierarchical network embedded in a viscous ground substance of negatively charged glycosaminoglycans (GAGs) and other proteins [2]. The collagen (types I and III) makes up 34 to 77% of the dry weight [5, 6], with evidence from human tissue studies showing that this dry weight content remains constant during gestation [7]. The cervical collagen fiber ultrastructure has been studied using X-ray diffraction [8, 9], magnetic resonance diffusion tensor imaging (MR DTI) [10], second harmonic generation imaging (SHG) [11, 12] and optical coherence tomography [13, 14]. In general, the collagen fiber network is reported to be anisotropic with different preferred orientations in distinct anatomical regions within the cervix. X-ray diffraction studies [9] found three radial zones of preferentially aligned collagen fibers, where collagen fibers along the outer edge and next to the inner canal predominately run parallel to the canal and collagen fibers in the mid-stromal area run circumferentially around the canal. The dispersion of these fibers was found to be aligned in a narrow range of 50 to 70 degrees within each zone. MR DTI [10] confirmed the inner and middle zones but the outer zone was not resolved. More recent preliminary SHG data from Feltovich *et al*. [15] and OCT studies from our group [14] reveal a large band of circumferential fibers extending to the outer edge of the tissue. To date, it is unclear how this ultrastructure evolves with pregnancy.

In this study, we use OCT [16], a non-invasive imaging technique based on the principle of low coherence interferometry, to image and characterize the cervical fiber ultrastructure. Using OCT, the sample morphological information in depth is obtained by interfering backscattered photons from a sample irradiated with a broadband low coherence source with a reference beam. A typical OCT system can achieve a high axial resolution at the micron level, a penetration depth up to 2 mm, and video rate data acquisition, and thus emerges as a promising image modality to image a variety of organ systems [17–20]. In particular, efforts have been made to image cervical tissues for cancer detection [21–23] by analyzing the layered structure of the epithelium, the basement membrane, and the stroma. In addition to cancerous structure, OCT can be also used to image the collagen fiber network. Our previous work has demonstrated the feasibility of imaging entire axial cervical slices [14]. This was enabled by an image-stitching algorithm to increase the field of view (FOV), encompassing entire axial slices (~3 cm × 3 cm), which allowed for assessment of collagen fiber orientation trends and for identification of unique anatomical regions. Our previous methods for fiber orientation estimation [24, 25] targeted extracting dominant fiber orientation in a subregion, however it could not provide detailed information about non-dominant orientations, resulting inaccuracy in the evaluation of fiber dispersion.

Building on our previous OCT investigation of the human cervix and the work of others, we report collagen fiber orientation maps of whole, unfixed, axial cross-section slices of non-pregnant and pregnant human cervical tissue to visualize anatomically-relevant trends. Due to the complex structural environment of the cervix, we hypothesize that the fiber orientation and local dispersion is heterogeneous with regions determined by anatomic location. We postulate that the homogeneity of fiber orientation and local dispersion will depend on four anatomic quadrants of the cervix (posterior, anterior, left and right) and will depend on the radial location from the inner canal. In this paper, we present the methodology of OCT imaging on human cervical tissue, and describe the fiber orientation maps and collagen fiber orientation distribution and dispersion across four quadrants and different radial distances from the inner canal.

## Methods

### Sample collection and preparation

Thirteen human cervices were collected from consented hysterectomy patients by an IRB approved protocol at Columbia University Medical Center (Table 1). Among the cervices, 11 were from non-pregnant (NP) patients undergoing hysterectomy for benign indications and 2 were from pregnant (PG) patients undergoing cesarean hysterectomy due to abnormal placentation. Patient age ranges from 36 to 49 and parity number from 0 to 5. The cervices were sliced perpendicular to the inner canal (Fig 1) immediately after hysterectomy using a custom-built slicer. The thickness of each slice was 3–5 mm. Axial slices within the upper half of cervix were excised. In this study, we analyzed the slice that is closes to uterus for each cervix. All samples were kept on dry ice and then stored at -80°C for later imaging. This study was approved by the Columbia University IRB, with an IRB protocol Number: IRB-AAAL4005. Study participants gave their consent by signing a written consent form that was approved by the Columbia University IRB. A more detailed protocol used for sample collection and preparation is described in our earlier work [26].

**Table 1:** Patient demographics of specimens used for this study.

Specimen Number	Age	Pregnancy Status	Gravidity / Parity	Obstetric History	Figure Number
1	42	NP	5/1041	1 VD, 3 VTOP	2
2	41	NP	6/4024	4 FT VD, 1 VTOP, 1 SAB	3
3	46	NP	0/0000	NA	4
4	40	NP	2/0020	VTOP, SAB	5A
5	43	NP	1/1001	VD	5B
6	49	NP	1/1001	VD	5C
7	46	NP	4/1031	VD, 3 VTOP	5D
8	40	NP	3/3003	3 VD	5E
9	48	NP	9/5045	5 FT VD, 4 VTOP	5F
10	36	NP	4/4004	4 CS	5G
11	46	NP	3/3003	VD, CS, VBAC	5H
12	30	PG	5/1031	CS, 3 SAB	5I
13	42	PG	5/2022	NA	5J

The first column relates patients with figure numbers of their OCT images. Gravidity is equivalent to the total number of pregnancies. Parity data is presented in TPAL recording system. TPAL stands for term, preterm, aborted, and living deliveries, corresponding respectively to each of the 4 digits. VD = vaginal delivery, VTOP = voluntary termination, FT = full term, SAB = spontaneous abortion (miscarriage), CS = cesarean section, VBAC = vaginal birth after cesarean, NA = not available.

### OCT scan and fiber recognition algorithm

Before OCT imaging, cervical slices were thawed in phosphate buffered saline (PBS) overnight at 4°C, and the surface closer to the internal os was microtomed. During the imaging procedure, the cervical slice was laid on top of a gauze soaked in PBS to keep the tissue hydrated. Samples were imaged using a commercial OCT system, Telesto I (Thorlab GmbH, Germany). The system is an InGaAs based system with its source centered at 1325 nm and a bandwidth of 150 nm. The axial and lateral resolutions are 6.5 μm and 15 μm in air, respectively. In our experiments, each volume consisted of 900 × 900 × 512 voxels, corresponding to a tissue volume of 4.5 mm × 4.5 mm × 2.51 mm (in air). Samples were placed in a linear translation stage underneath the objective. For each sample, we obtained multiple volumes. There was an overlap proportion of at least 10% between two adjacent volumes. A white light camera obtained an image of the sample corresponding to the OCT FOV. The camera images and OCT images were calibrated by the default factory setting.

Volumetric data was stitched based on the shift invariant feature in camera image within the *en face* plane and surface information of the OCT data in the axial direction [14]. Upon generating the three dimensional data, parallel *en face* images were obtained 245 μm beneath the surface to perform 2D fiber directionality and dispersion analysis [14]. Fiber orientations were extracted for each pixel (5 μm × 5 μm) by optimizing a pixel-wise fiber orientation method [27, 28] for OCT image datasets. In each *en face* image, the collagen fiber region was masked based on the signal to noise ratio. Then, the image was enhanced through histogram stretching. The image was sharpened by second order Butterworth high pass filter and subsequently denoised by a median filter. A weighted summation scheme was utilized to determine the fiber orientation at each pixel over the entire region. For a pixel of interest, *p*_0_, there were multiple candidate directions *α*_j_ towards its neighboring pixels, *p*_1_ and *p*_2._ A weight was assigned to each candidate direction as following:

$$w_{j}=w_{i}\times w_{d}$$ (1)

$$w_{i}=\frac{1}{3}-std\left(p_{1},p_{0},p_{2}\right)$$ (2)

$$w_{d}=\frac{1}{dist(p_{0},p_{2}\;or\;p_{1})}$$ (3)

The weight was determined by two factors, *w*_*i*_ and *w*_*d*_. The first factor, *w*_*i*_, was the intensity variations between the pixel of interest (*p*_*0*_) and its neighboring pixels (*p*_*1*_ and *p*_*2*_) along a particular direction. The second factor, *w*_*d*_, was the corresponding distance between the pixel of interest (*p*_*0*_) and the neighboring pixel (*p*_*1*_ or *p*_*2*_). The direction, *α*, of target pixel *p*_0_ is determined by the weighted circular mean of all direction candidates as described in Eq 4:

$$\alpha =arg(\sum_{j=1}^{N}w_{j}\times exp(i\alpha_{j}))$$ (4)

Where N is the number of direction candidates around pixel *p*_0_. Given the direction information of each pixel, we generate the directionality map of the whole OCT image.

**Fig 2:**
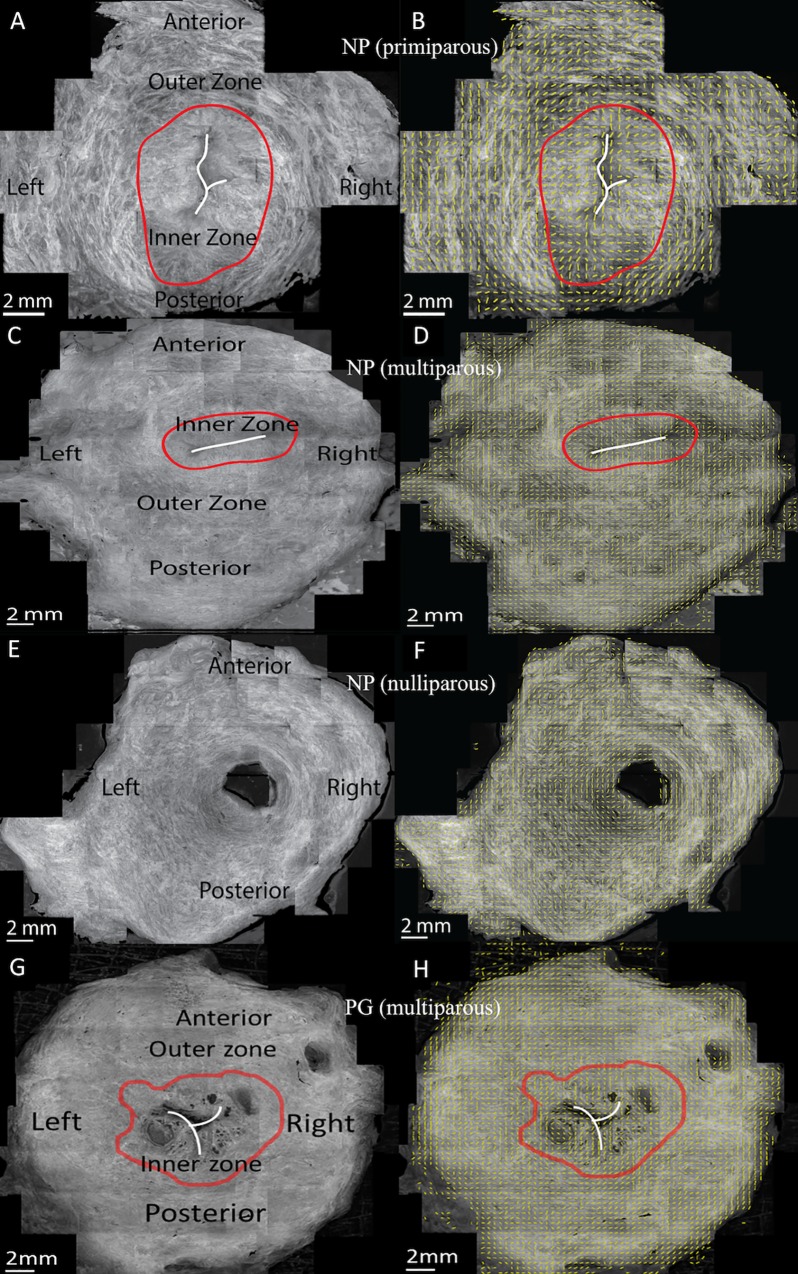
Representative OCT *en face* images and overlaid fiber orientation maps of cervical slices with different inner zone widths. (A), (C), (E), and (G) are OCT *en face* images taken 245 μm beneath the cut surface and (B), (D), (F), and (H) are overlaid fiber orientation maps. (A), (C), and (E) were from non-pregnant patients with wide, narrow, and no inner zones. (G) was from a pregnant patient. The white line sketches the inner canal. The red contour delineates the inner radial zone according to local fiber orientation. The yellow bars in (B), (D), (F), and (H) show local dominant fiber orientation in each 400 × 400 μm sub-region. (A)-(B), (C)-(D), (E)-(F), and (G)-(H) are Speicemen 1, Speciemen 2, Speciemen 3, and Speciemen 13 in Table 1 respectively.

Based on the pixel-wise orientation information, we obtained the directionality map of collagen fibers within the *en face* image (Fig 2A). The directionality map was further divided into sub-regions of 400 μm × 400 μm along the radial direction in the four anatomical quadrants from inner canal to outer edge (Fig 2A). In each 400 μm × 400 μm subregion, a 2D von-Mises probability density function,

$$P(x)=\frac{e^{b\cos\left(x-\theta \right)}}{2\pi I_{0}(b)},$$ (5)

was fit to the pixel-wise orientation data to determine the fiber direction *θ* and the concentration parameter *b*. The two parameters were estimated by a least squares method using MATLAB (MathWorks, R2014b) function (fit). *I*_0_(*b*) is a modified Bessel function of the first kind of order 0. Here, *θ* ∈ [0,2*π*) is the dominant fiber direction and *b* > 0 is the concentration parameter [29]. The concentration parameter *b* describes the dispersion level of Von Mises distribution. When *b* approaches 0, the distribution gets closer to isotropic (circular in 2D case), and as *b* increases to infinity the distribution gets closer to perfectly aligned fibers. In other words, *b* is inversely related to fiber dispersion where a low *b* describes a high fiber dispersion and a high b describes a low fiber dispersion.

### Statistical analysis

A group of analysis of variance (ANOVA) tests were performed in MATLAB using one-way ANOVA function (anova1) and multiple comparison function (mulitcompare) to compare the von-Mises fiber ultrastructure parameters (b and θ) between NP and PG specimens and among NP specimens with different parity. The data normality was verified by Kolmogorov–Smirnov test in MATLAB (kstest function) before performing the ANOVA analysis. The homogeneity of these fiber ultrastructural parameters within individual sample slices were assessed by comparing results between circumferential quadrants, inner and outer radial zones (Fig 2A).

In the circumferential direction, the cervical slice was divided into four anatomical quadrants. In the radial direction, the cervical slice was divided into inner and outer zones. The border between the inner and outer radial zones was manually determined by differentiating the distinct patterns of fiber orientation of the two zones. The radial direction was also subdivided into 400 μm × 400 μm subregions as described by the pixel-wise fiber tracking method above. Parity is the number of times that a woman has given birth. We divided our patients into three parity groups: nulliparous patients (*n* = 2) who have never given birth, primiparous patients (*n* = 4) who have given birth once, and multiparous patients (*n* = 5) who have given birth two or more times.

When *b* and *θ* were compared between different samples, averages were taken of the results from all 400 μm × 400 μm subregions within the quadrant and radial zone. When comparing *b* and *θ*, the variance of *b* and *θ* along radial direction within each quadrant and zone were also measured by calculating the standard deviation. All ANOVA tests were performed in MATLAB using the anova1 function where a *p*-value of 0.05 was considered statistically significant.

## Results

### OCT *en face* images and fiber orientation maps

The regional collagen fiber architecture of the upper half of the human cervix is depicted in 2D image a fixed axial distance below the surface in Fig 2 using the method in [24, 30]. In 9 NP tissue samples out of the 11 NP samples imaged, two radially zones are found with distinct fiber orientation characteristics (Fig 2 and S1 Fig). In these tissue slices there is an inner zone with collagen fibers preferentially aligned in the radial direction and an outer zone with collagen fibers preferentially aligned in the circumferential direction. The shape of the inner zone and the fiber orientations in this zone are highly affected by the shape of the inner canal. The inner zone can be relatively wide or narrow, where Fig 2A and 2B shows the widest inner zone which is around 30% of the slice radius and Fig 2C and 2D shows a narrower inner zone. In the 4 remaining slices, including both pregnant samples, there is no inner zone and the whole slice is dominated by circumferentially aligned fibers (Fig 2E and 2F). For many slices (Fig 2C and 2D), S1A, S1B, S1E, S1F, S1H and S1I Fig), the inner canal opening aligns from left to right. Others have the inner canal opening aligning from anterior to posterior, or the inner canal is round or has an irregular shape. Based on the samples we examined, there is no clear relationship between the inner zone size and patient parity.

**Fig 3:**
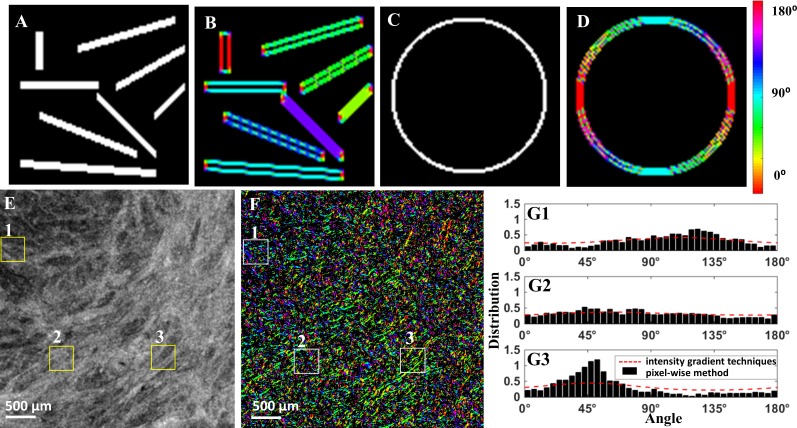
(A), (C) Synthetic data for algorithm validation; (B), (D) processed data with pixel-wise fiber orientation. (E) Original OCT image from an *en face* plane (Specimen 8 in Table 1); (F) pixel-wise directionality map; (G1)-(G3) Histogram of orientation obtained from pixel-wise fiber orientation method in sub-regions from 1 to 3. Distribution of fiber orientation in the three regions using pixel-wise fiber orientation method and intensity gradient techniques [14, 15]. Each box is 400 μm × 400 μm.

We validated our pixel-wise fiber recognition algorithm on synthetic data in Fig 3, where our algorithm accurately estimates the directionality of segments oriented at various orientations (A-B) and circular shape (C-D). The new pixel-wise fiber recognition algorithm is superior to the gradient-based method because the pixel-wise method is able to capture the existence of distinct fiber families at different orientations, especially non-dominant orientations. A directionality map using the pixel-wise fiber orientation algorithm of a cervical sample is shown in Fig 3F, in comparison with the OCT *en face* image in Fig 3E. The fiber distribution obtained from three subregions using the pixel-wise method and the gradient-based method in [24, 25] were compared in Fig 3G. In general, the estimated dominant direction using two methods approximate each other within each subregion. However, the gradient method is unable to capture the actual fiber distribution of the probability of fiber existence at each angle.

**Fig 4:**
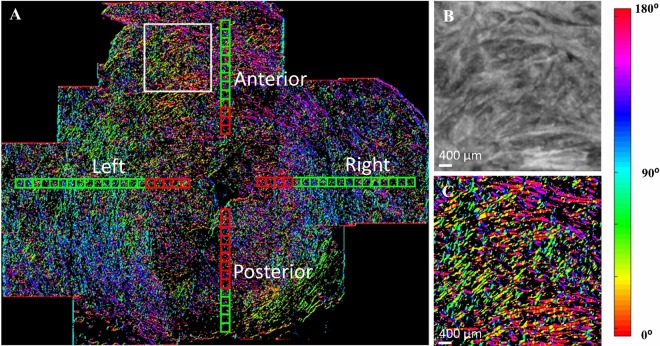
A pixel-wise directionality map on an *en face* image parallel from and 245 μm beneath the cut surface (Specimen 1 in Table 1). (A) directionality map with locations of 400 μm × 400 μm subregions corresponding to 80 pixels × 80 pixels.; (B) OCT image within the white box in (A); (C) directionality map within the white box in (B). Pixels with no fiber information are coded in black. Each 400 μm × 400 μm subregion represents a location for the fiber orientation and dispersion analysis in the A (anterior), P (posterior), L (left), and R (right) quadrants. Along the radial direction, the boxes are divided into inner region (red) and outer region (green).

A typical example of the pixel-wise method on a stitched OCT cervix image comprised of 24 OCT volumes is shown in Fig 4. The original OCT image is an *en face* image 245 μm parallel to the cut surface as shown in Fig 2A. From the pixel-wise directionality map, such as Fig 4A, we observe a circumferential trend of fiber in the outer zone. From a zoom-in box in Fig 4B and 4C corresponding to a 4 × 4mm region, it shows fiber directions can vary dramatically within a small region. Similar circumferential trends and direction variation patterns are observed in all other cervical samples.

**Fig 5:**
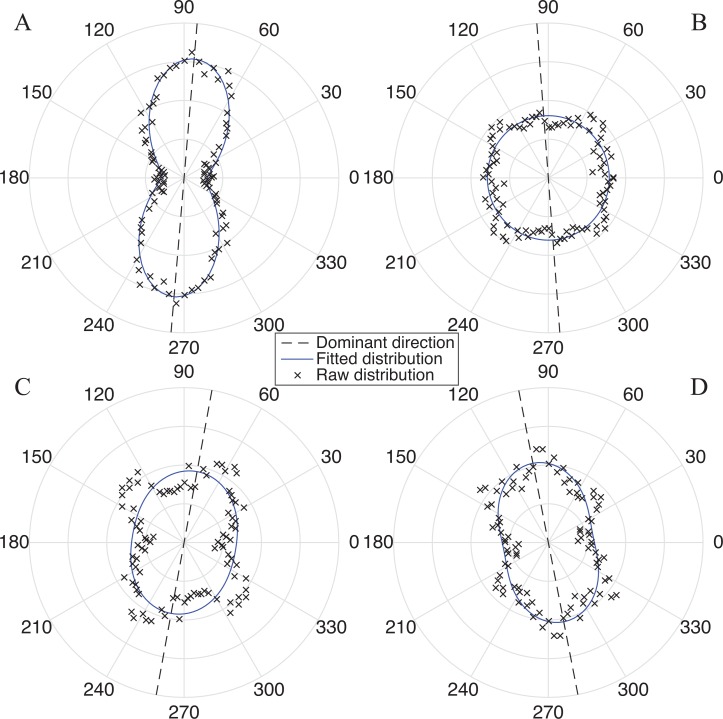
Representative fiber distributions found in the upper cervix and corresponding 2D von-Mises fits. The dominant direction *θ* is shown by dotted line. All four subregions are taken from the outer radial zone of the same NP sample (Specimen 5 in Table 1). A subregion with (A) a single family of fibers that have the most alignment (*b* = 0.820) and (B) highly dispersed fibers that are randomly oriented in the plane. A subregion with (C) two fiber families and (D) three fiber families. (Note: current distribution fitting methodology cannot distinguish the multiple fiber families.)

2D von-Mises distribution provides a close fit to the raw fiber dispersion data. Concentration parameter *b* can be as high as 0.820 and as low as 0.010 as shown in Fig 5A and 5B. For certain subregions, more than one family of fibers can be observed where the current 2D von-Mises analysis cannot capture these distinct fiber families (Fig 5C and 5D). The fitting for multiple families of fibers will be discussed in discussion.

### The posterior and anterior cervix contains regions of preferentially-aligned collagen fibers

**Fig 6:**
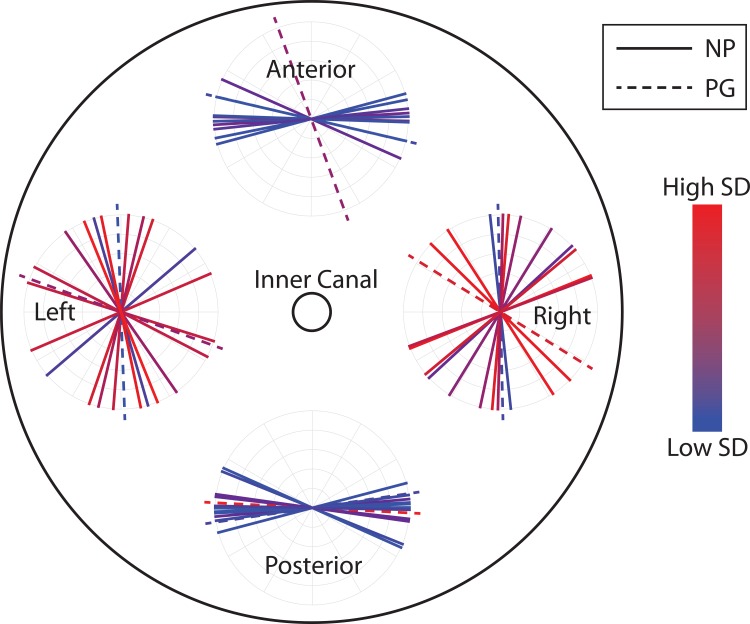
The dominant fiber direction *θ* in the outer radial zone in four quadrants for all specimens imaged. Each circle represents the dominant fiber directions in one quadrant. Each line represents one cervical sample averaged across all 400 μm × 400 μm radial subregions, with the line color representing the standard deviation (SD) between the radial subregions. Red represents higher SD and blue represents lower SD. Posterior and anterior quadrants both have more uniform dominant directions among samples (lines having a narrower spread) and within a single specimen there is lower SD between the radial subregions (lines having a bluish color). Left and right quadrants have a wider spread of the dominant fiber direction between samples, and within an individual sample, fiber directions also change more dramatically along radial direction.

The upper half of the cervix contains zones of preferentially-aligned collagen with distinct fiber directionality and dispersion properties in the posterior, anterior, left, and right quadrants (Fig 6). The dominant fiber directionality data *θ* for each of the 13 specimens averaged across radial subregions in the outer radial zone within each anatomical quadrant are represented in Fig 6. For all 13 specimens, including NP and PG, the dominant fiber directionality in the posterior and anterior quadrants of the cervix is in the circumferential direction, with fibers circling around the inner canal. When comparing these anterior and posterior quadrants between specimens, the averaged directions *θ* are within a small range (≈35°) with one exception of the anterior of one PG sample. In the left and right quadrants, although the average dominant direction for all specimens is circumferential, dominant directions themselves are scattered within a larger range (≈140°), which indicates a higher variability between specimens in the left and right quadrants.

**Fig 7:**
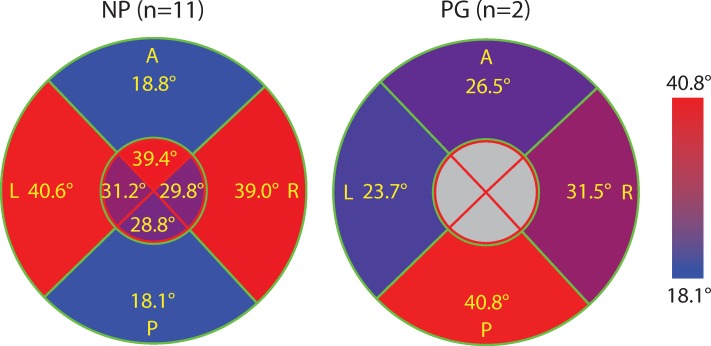
The average of the SD of dominant fiber direction *θ* between radial subregions for NP and PG specimens. Values are shown in eight regions that include the inner and outer radial zones of four quadrants. Each region is color-coded such that red represents higher SD (more heterogeneous *θ* between radial subregions) and blue represents lower SD (more homogeneous *θ* between radial subregions). The anterior and posterior quadrants of the NP cervical tissue samples have more homogeneous circumferential fibers compared to its left and right quadrants.

The data normality is verified in every radial quadrant and inner/outer region since Kolmogorov-Smirnov test results accept the null hypothesis (lowest p = 0.28). When comparing 400 μm × 400 μm radial subregions within a single slice in the outer zone for NP specimens, the standard deviation (SD) of the dominant direction *θ* is higher in the left and right quadrants comparing to that in posterior and anterior quadrants (Fig 7). This higher SD means fiber changes orientation more dramatically along radial direction and the tissue is more heterogeneous. This difference is very significant in the outer zone (*p* < 0.001) but not significant in the inner zone (*p* > 0.983). The SD of *θ* in the outer zone of posterior and anterior quadrants of NP samples is different than the rest regions in NP samples as well as all regions in PG samples (S2A Fig). If we group them this way (S2C Fig), the difference is very significant (*p* = 3.4 × 10^−6^).

### The posterior and anterior of the outer radial zone of NP specimens have the lowest collagen dispersion

**Fig 8:**
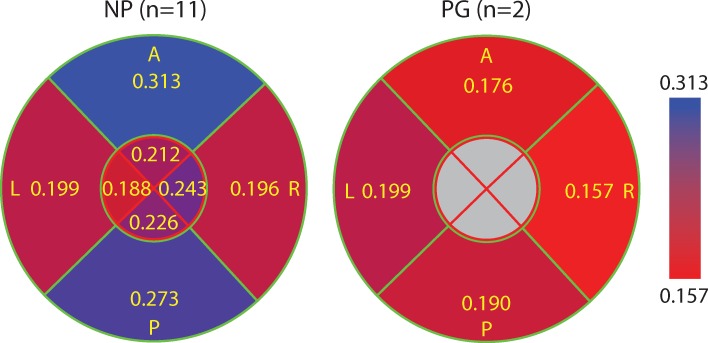
The concentration parameter *b* averaged for all 400 μm × 400 μm radial subregions in each quadrant for all NP and PG specimens. This figure is color-coded such that blue represents higher *b* (lower dispersion and more aligned fibers) and red represents lower *b* (more dispersed and randomly oriented fibers). Overall, the collagen fibers of the NP cervices had tighter aligned fibers within the 400 μm × 400 μm subregions compared to the PG samples. Within the NP samples, the A/P quadrants in the outer zone have the most aligned fibers within the 400 μm × 400 μm radial subregions compared to the rest.

**Fig 9:**
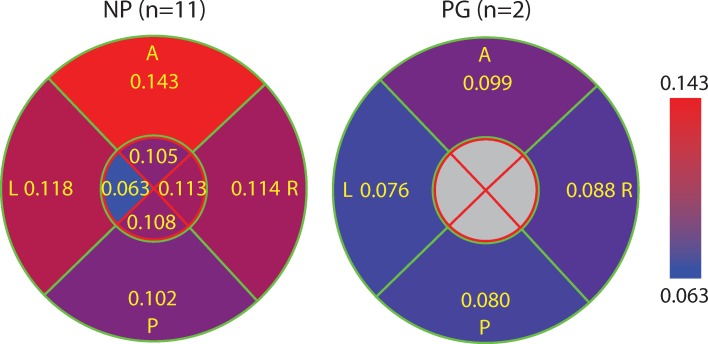
The average of SD of the concentration parameter *b* between different radial subregion. This figure is color-coded such that red represents higher SD (relative heterogeneity of *b* along radial direction) and blue represents lower SD (relative homogeneity of *b* along radial direction). The highest value appears in the A quadrant of NP samples and the lower values appear in the L quadrant of NP samples and P/L quadrants of PG samples.

The posterior and anterior quadrants of the outer zone in NP specimens have the lowest fiber dispersion in the 400 μm × 400 μm subregions (i.e. highest concentration parameter *b*, Fig 8) compared to other quadrants in NP samples and to all quadrants in the PG samples. The left and right quadrants of the NP samples have similar fiber dispersion properties compared to all quadrants of the PG specimens (Fig 8). Similar to the SD of *θ*, if we group the outer zone of posterior and anterior of NP samples together and group the remaining regions in NP samples as well all regions in PG samples together, the difference in *b* is statistically significant (*p* = 2.1 × 10^−7^, see S3 Fig). Within the PG samples there is no significant difference among quadrants. The variance in fiber dispersion, represented by the SD of concentration parameter *b*, between 400 μm × 400 μm radial subregions within a single slice are not significantly different (Fig 9).

### There is difference in dispersion between NP and PG but no difference is found between NP samples with different parity

**Fig 10:**
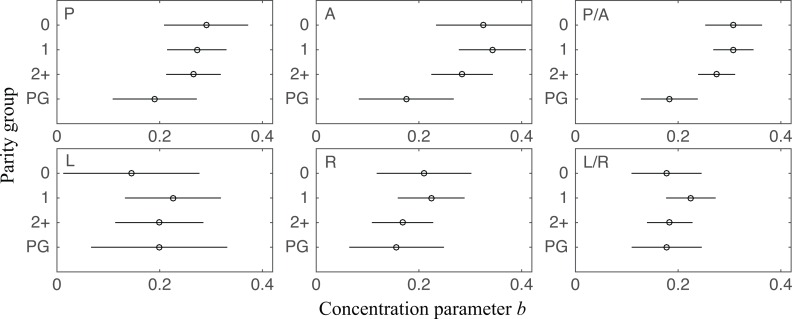
Concentration parameter *b* in different parity groups and pregnancy statuses. Four groups are nulliparous NP (group “0”, *n* = 2), primiparous NP (group “1”, *n* = 4), multiparous NP (group “2+”, *n* = 5), and PG (group “PG”, *n* = 2). Results from each quadrant in the outer radial zone and some combinations are shown. There is no significant difference between nulliparous NP, primiparous NP, and multiparous NP. In quadrants P/A, PG has a lower *b* than NP groups. This result is significant when we analyze the data from both quadrants combined (*p* between 0.006 and 0.045). No significant difference is found in L/R quadrants.

**Fig 11:**
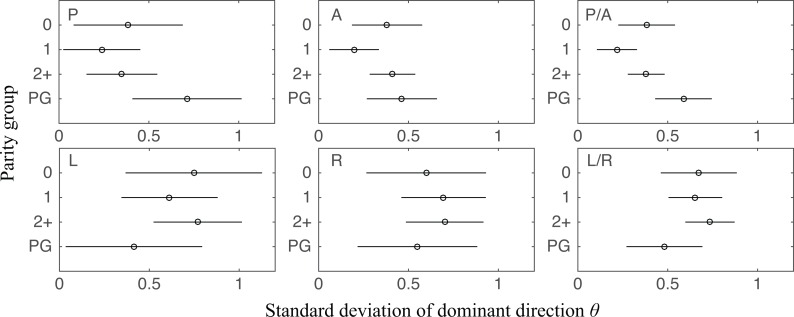
SD of dominant direction *θ* (unit: rad) in different parity groups and pregnancy statuses. Results from each quadrant in the outer radial zone and some combinations are shown. NP groups do not have significant differences among themselves. Between NP groups and PG, the only significant difference is found between primiparous NP and PG when P/A quadrants are combined (*p* = 0.004). Unlike the analysis for *b*, nulliparous NP (*p* = 0.276) and multiparous NP (*p* = 0.143) do not have a significant difference with PG in the SD of *θ*. No significant difference is found in L/R quadrants.

We found a statistically significant difference in *b* between NP and PG specimens in posterior and anterior in the outer zone (Fig 10). In further detail, among four quadrants, *b* of PG specimens has distinctly lower mean values in posterior and anterior than NP specimens, which suggests PG specimens have more dispersed collagen fibers. The difference is significant if we compare the combined posterior and anterior region and combined left and right regions (*p* between 0.006 and 0.045). Such difference is not found with the variance of *θ* (Fig 11). Among parity groups in NP specimens, there is no difference found for either *b* or *θ* (Figs 10 and 11).

## Discussion

In this paper, we present a regional OCT collagen fiber orientation and dispersion analysis of 13 fresh, unfixed human cervical slices. To measure local fiber orientation and dispersion, a new pixel-wise fiber orientation algorithm is developed for cervical tissue analysis. Based on this method, fiber orientation maps are generated to visualize and measure the tissue-level architecture of the upper cervix. In all of the cervical fiber orientation maps, there is a dominant outer radial zone of preferentially aligned collagen fibers circling around the inner canal where the posterior and anterior quadrants are more aligned than left and right quadrants. In 9 out of the 11 non-pregnant samples, there is an additional inner radial zone with fibers preferentially aligned in the radial direction that is perpendicular to inner canal opening direction. In this inner radial zone, though, the trends are difficult to study because this zone also includes mucous glands located around the inner canal opening, which cannot be differentiated from dense collagen fibers.

**Fig 12:**
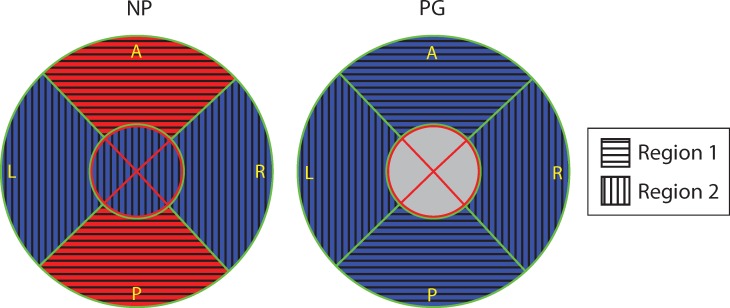
Definition of Region 1 and Region 2. The collagen fiber network in cervical tissue is not homogenous and it has two distinct regions: Region 1, the P/A quadrants in the outer radial zone (horizontal stripe), and Region 2, the rest (vertical stripe).

The NP cervical tissue samples measured in this study have two regions with distinct fiber directionality and dispersion properties. The posterior and anterior of the outer zone is labeled Region 1 and the remaining parts of the cervix (left and right of outer zone and all inner zones) are labeled Region 2 (Fig 12). For a NP cervix, Region 1 and Region 2 have different fiber dispersions between Regions and similar dispersions within each Region. However, when a NP cervix becomes a PG cervix, Region 1 will have a shift in the fiber dispersions so that the properties are similar to Region 2 while Region 2’s properties do not shift. In other words, Region 1 is more sensitive to pregnancy status and remodels more dramatically than that happened in Region 2 during pregnancy. The arguments above are verified by ANOVA test in Result section by comparing Region 1 in NP with Region 2 in NP and all Regions in PG (S2C Fig and S3C Fig).

We believe the regional differences in collagen fiber properties within a single sample and between samples are influenced by the anatomical and loading environment of the cervix in the pelvic region. The cervix is the lower portion of uterus. The upper portion of the cervix, or the portio supravaginalis, lies above the vaginal attachment to the cervix. Cardinal ligaments attach this portion laterally (i.e. left and right), and the bladder lies anterior to the cervix separated by loose connective tissue. In pregnancy, the upper cervix is substantially loaded by the growing fetus [4]. The positioning, symmetry, and shape of the uterus and cervix drive the patterns of cervical stress and stretch and can be vastly different for each person [31]. Often in pregnancy the cervical axis is angled posteriorly from the uterine axis. This positioning leads to increased tissue loads and stretching in the anterior and posterior sections of the cervix [4]. The angle of the cervix with the uterus can be a potential cause of the increased anisotropy in Region 1 of the cervix, and the fact anatomical factors vary widely between patients can explain the variability between samples. Related research [32] of finite element analysis of human uterus and cervix also supports the heterogeneity of fiber dispersion we find between quadrants. The FEA analysis demonstrates that the collagen directionality and dispersion play a role in resisting physiological relevant deformation during pregnancy. Further studies with larger patient populations must be conducted to understand the mechanical loads on the cervix and cervical tissue remodeling behaviors during pregnancy.

Many imaging modalities that have been used to study collagen fiber orientation and dispersion of hydrated soft tissues, with each method suited for different length scales and tissue sample preparations. Our OCT analysis presented here interrogates the tissue within 400 μm × 400 μm subregions across whole, hydrated, and unfixed axial tissue slices. In comparison, second-harmonic generation (SHG) microscopy is a standard method for the characterization of hydrated biological tissue at sub-cellular resolution. The FOV of SHG systems can be tens of microns to hundreds of microns, with a resolution of better than 2μm [33]. With these features, SHG captures information on fibril-level structure and the “crimping” or waviness of fibrils. Small angle light scattering (SALS) uses laser light to image fiber orientation and dispersion [34]. Collagen fibers can be distinguished from striated muscle, smooth muscle, and elastic fibers from the resulting angular distribution of scattered light because striated muscle, smooth muscle, and elastic fibers have different birefringence properties [35–37]. Each reading covers ~ 4 × 4 μm area which is the size of a single or small group of collagen fibers. In the future, functional extensions of OCT can also allow for interrogating the anisotropy and birefringence properties of cervical samples using polarization sensitive OCT [23, 38, 39].

We carefully compared our conclusion with a similar paper, [9], which used x-ray diffraction to study the collagen ultrastructure in human cervix. The study presents detailed cervical collagen orientation and dispersion data from NP human 1 mm^3^ cube samples that have been scanned in three orthogonal directions. There are similarities and differences between this X-ray diffraction study and our OCT study. First, they found that near the internal os there are three zones of preferentially aligned collagen fibers—the inner zone, the middle zone, and the outer zone. The X-ray data found that the both inner and outer zone have longitudinally aligned fibers while the middle zone has circumferentially aligned fibers. Our study indicates a similar inner zone. Since we cut and scan axial slices, it is not possible to see longitudinal fibers in our OCT images. It may be possible that the radially aligned fibers seen in the OCT data are skewed longitudinal fibers that not perfectly aligned with the inner canal. The middle zone indicated in the X-ray study is similar to our outer radial zone of circumferential fibers. However, our OCT images show these circumferential fibers extend to the outer edge of the sample. They also measured the radial thickness of each zone, the inner and outer zones are 3–5 mm and the middle zone is 5–12 mm. The inner zone in our study ranges from 0 mm to 6 mm and the middle zone ranges from 6 mm to 14 mm, which is comparable to the results presented by them. Second, the fiber dispersion in [9] is much tighter than that in our study. In [9]’s finding, almost all the fiber are aligned within 90° while in our study there are always fibers throughout 180°. We believe reason for the difference lies in the difference of method including sample preparation (our samples were not fixed) and fiber recognition.

This research presented in this paper has the following limitations. First, as discussed earlier, the collagen fiber network is three-dimensional but fiber orientation and dispersion were only studied in two dimensions. Longitudinal fibers cannot be verified in this research because OCT images were stitched in the plane that is perpendicular to the inner canal. Second, only the available slice that is closest to the internal os had been studied. We selected the first slice to start our research because the internal os is the location of premature funneling [32] and maximum stress [4] during pregnancy. The premature funneling is often followed by opening up from the rest of the cervical inner canal and preterm birth. As we found in different quadrants, it is highly possible that the cervix is heterogeneous in the longitudinal direction since the percentage and type of biological and chemical components have been found to be different along longitudinal direction [5] and the inner zone was found to disappear as we approach to the external os [9]. Third, due to the limited number consented patients, we have a smaller database comparing to research that uses animal tissue. Currently, our preliminary study on pregnancy is based on 2 specimens. We plan to collect more specimens and draw a more conclusive comparison in our future study. Also, we will keep increasing the number within nulliparous, primiparous, and multiparous groups. Fourth, PG slices were not necessarily at the upper cervix because both of our PG samples were from patients with accreta and it is difficult to distinguish the location of the internal os. In order to avoid tissue with accreta, cervical slices of PG patients were obtained at the most proximal location available so the slices could be from mid-cervix. Fifth, although our pixel-wise analysis can capture the patterns of multiple fiber families, the von-Mises based distribution fitting is not efficient for subregions with two dominant families. Since the only case (two families) that cannot be efficiently captured by von-Mises distribution accounts for about 5% of all subregions, von-Mises is a good distribution model overall. In the future, we will improve our method and develop a more generalized method for all fiber family patterns.

## Conclusions

In this study, we measured the heterogeneity of local fiber orientation and dispersion in human tissue slices from the upper cervix using an OCT pixel-wise fiber orientation algorithm. We found that human cervical tissue has a distinct collagen fiber ultrastructure where collagen fiber orientation and dispersion vary according to anatomical quadrants. We found that in non-pregnant tissue, the anterior and posterior quadrants have highly aligned circumferential collagen fibers that are less dispersed than the left and right quadrant. Overall, we found that the non-pregnant samples examined here had more aligned and less dispersed collagen fibers than pregnant tissue, and that there was no difference in collagen properties between non-pregnant samples of different parity. The OCT imaging and tracking algorithm presented here is suited for our application because it offers tissue fiber ultrastructure characteristics at a length scale appropriate for implementation into a previously developed fiber-based continuum material model for human cervical tissue [32]. Additionally, the whole sample fiber maps inform the implementation of tissue architecture into large-scale finite element models of pregnancy. Lastly, OCT is a nondestructive technique, which allows for ultrastructural, biochemical, and mechanical analysis to be conducted on a single sample. In future work, multiple slices from internal os to external os will be analyzed to look for trend of fiber dispersion along longitudinal direction, mechanical tests will be conducted to determine corresponding material behavior, and the structural importance of the regional ultrastructural properties of the cervix will be explored in finite element models of human pregnancy.

## Supporting Information

S1 Fig OCT *en face* images of cervical slices in addition to those shown in Figs 2–4 for (A)-(H) NP (Specimen 4–11 in Table 1) and (I) PG (Specimen 12 in Table 1) specimens. For the NP specimens, the label in the parenthesis represents the parity groups or pregnancy status: “0” represents nulliparous, “1” represents primiparous, and “2+” represents multiparous. The orientation of the slice is shown in the legend.
(TIF)

S2 Fig Mean (circle) and comparison interval (bar) of the SD of dominant direction *θ* in different regions (unit: rad). In (A) all the quadrants are compared (inner and outer radial zones of NP samples; outer radial zone of PG samples). We can see that NP-outer-P/A has lower means than other groups. NP-outer-L/R, NP-inner-P/A/L/R, and PG-outer-P/A/L/R have similar means but the PG-outer-P/A/L/R have wider comparison intervals (higher variances). To better visualize the results and improve statistical quality, we further group the data as shown in (B). Among the four groups, NP-outer-P/A has lower means than other three groups but only two of them are significant different (comparison intervals do not overlap with each other). This can be further verified by (C) in which NP-outer-P/A is compared with the rest. A significant difference between the two groups (1) NP-outer-P/A has distinctly lower SD of *θ* than the rest of the slice and (2) NP-outer-P/A reforms and the fiber distribution becomes similar to the rest of the slice during pregnancy. This suggests fibers are, in organ scale, better aligned in NP-outer-P/A and this distinction disappears during pregnancy. Tests similar to S3 Fig were performed for SD of *θ*. Similar conclusions can be drawn that NP-outer-P/A has significantly lower SD of *θ* along radial direction than the rest which means the fibers are, in organ scale, better aligned in these regions and this distinction disappears during pregnancy. The only noticeable difference with *b* here is that the SD of *θ* in PG-outer group in (B) is large and the difference between it and NP-outer-P/A is not statistically different (comparison intervals overlap with each other).
(EPS)

S3 Fig Mean (circle) and comparison interval (bar) of concentration parameter *b* in different regions. Tests similar to S2 Fig were performed for *b*. Similar conclusions can be drawn that NP-outer-P/A has significantly higher *b* than the rest which means the fibers are, in organ scale, better aligned in these regions and this distinction disappears during pregnancy.
(EPS)
