# Supplementary figures and images for: Collagen Fiber Orientation and Dispersion in the Upper Cervix of Non-Pregnant and Pregnant Women
Source: PLoS One. 2016 Nov 29;11(11):e0166709. doi: 10.1371/journal.pone.0166709 (PMC5127549; doi:10.1371/journal.pone.0166709)

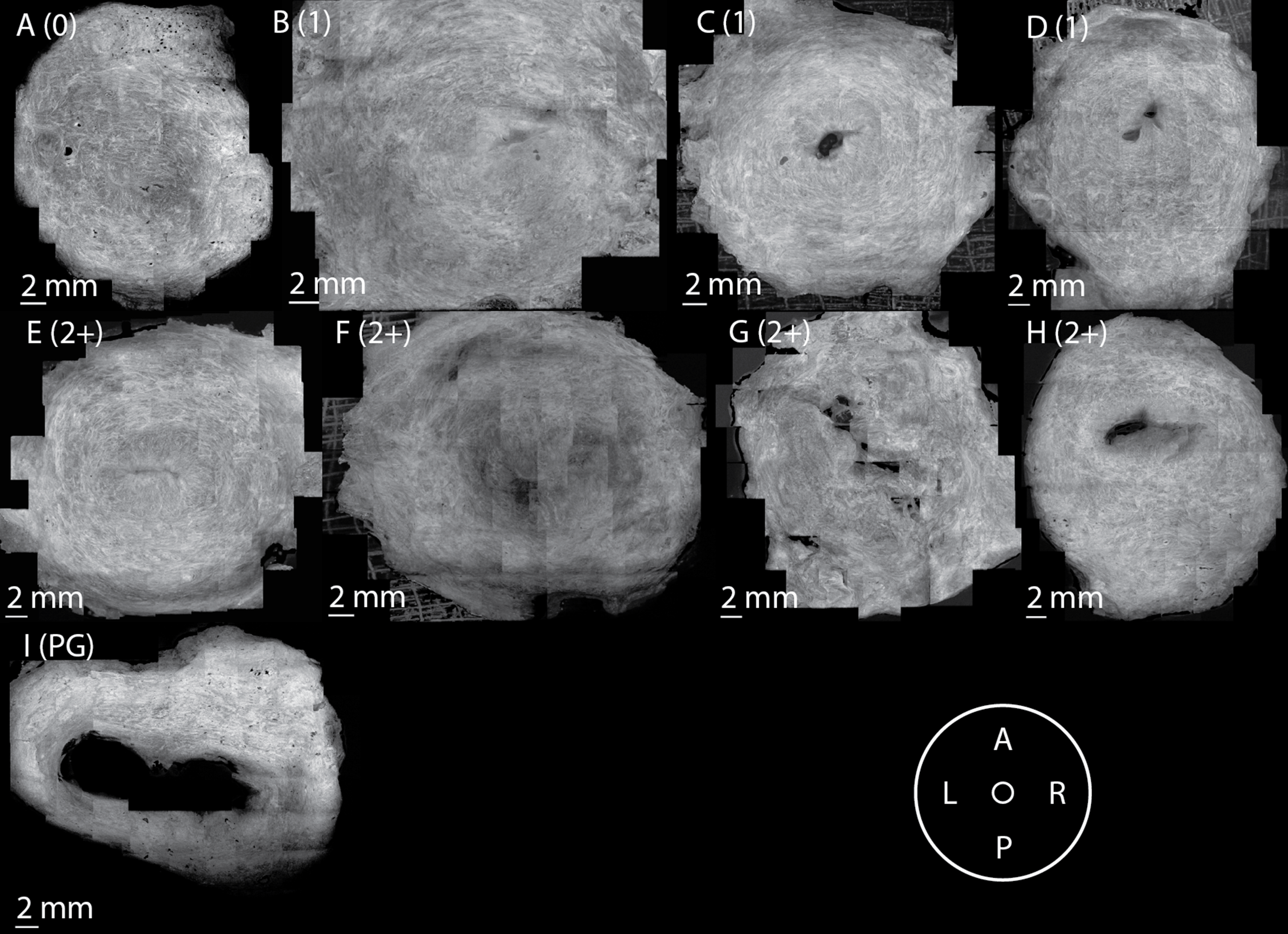

Supplement: S1 Fig — OCT en face images of cervical slices in addition to those shown in Figs 2–4 for (A)-(H) NP (Specimen 4–11 in Table 1) and (I) PG (Specimen 12 in Table 1) specimens. For the NP specimens, the label in the parenthesis represents the parity groups or pregnancy status: “0” represents nulliparous, “1” represents primiparous, and “2+” represents multiparous. The orientation of the slice is shown in the legend. (TIF) [file pone.0166709.s001.tif]
